# Periodontitis and Acute Myocardial Infarction Among Patients Visiting a Tertiary Care Center in Koshi Province of Nepal: A Case–Control Study

**DOI:** 10.1155/ijod/1143628

**Published:** 2026-01-24

**Authors:** Binita Limbu, Ashish Shrestha, Tarakant Bhagat, Santosh Kumari Agrawal, Naveen Kumar Pandey

**Affiliations:** ^1^ Department of Public Health Dentistry, College of Dental Surgery, B. P. Koirala Institute of Health Sciences, Dharan, Nepal, bpkihs.edu; ^2^ Department of Cardiology, B. P. Koirala Institute of Health Sciences, Dharan, Nepal, bpkihs.edu

**Keywords:** cardiovascular disease, case–control study, myocardial infarction, periodontitis, risk factors

## Abstract

**Background:**

Acute myocardial infarction (AMI) is the leading cardiovascular cause of morbidity and mortality worldwide. Atherosclerosis and acute thromboembolic events have been found to be related to chronic dental infections. But the evidence of the causal association of periodontitis and AMI is conflicting. This study was conducted to assess the association between chronic periodontitis and AMI among patients visiting a tertiary care center in Koshi Province of Nepal.

**Methods:**

A case–control study was conducted among 37 cases of AMI admitted to cardiology ward of B.P. Koirala Institute of Health Sciences (BPKIHS) and 37 controls from the relatives of the patients with no history of myocardial infarction. Data on sociodemographic characteristics, personal habits, and medical history was collected using a structured questionnaire. Blood pressure and BMI of each participant were measured. Medical data of patients were extracted from the patient files, and for controls, blood tests were done to determine random blood glucose and cholesterol levels. Oral examination was done to assess oral hygiene status, number of teeth present, and periodontal status. Multivariate logistic regression analysis used two models to adjust for the confounding variables. The level of significance was set at *p*  < 0.05.

**Results:**

Lower number of teeth and periodontitis were associated with an increased risk of having AMI with an odds ratio (OR) of 0.89; 95% CI: 0.82–0.97; *p* = 0.006 and OR = 2.85; 95% CI: 1.08–7.52; *p* = 0.034, respectively. A significant association was found between AMI and the presence of a lower number of teeth in the confounding factors‐adjusted model. The significant association between periodontitis and myocardial infarction was lost in both Models I and II after adjusting for other risk factors (adjusted OR = 1.49; 95% CI: 0.45–4.96; *p* = 0.515 and OR = 1.39; 95% CI: 0.37–5.29; *p* = 0.63, respectively).

**Conclusion:**

This study did not find significant association between periodontitis and AMI on adjusting for other risk factors. However, the crude marker of periodontitis, lower number of teeth present, was associated with the higher risk of AMI. Further studies are required to fully understand the nature of association of periodontitis and AMI.

## 1. Introduction

Cardiovascular disease (CVD) is the top cause of death and morbidity among the noncommunicable diseases globally [1]. It poses a significant health challenge worldwide and is responsible for 28.6% of all global deaths, with half attributed to ischemic heart disease. Analysis of global burden of disease data in 2021 showed that CVDs account for 22% of total deaths and 12.2% of total disability‐adjusted life years (DALYs) in Nepal, where ischemic heart disease is one of the prominent cause contributing to 10.9% of total deaths and 5.9% of total DALYs [2]. Myocardial infarction, also known as “heart attack,” is one of the main manifestations of ischemic heart disease, involving myocardial cell necrosis due to significant and prolonged ischemia. It occurs due to atherosclerotic plaque rupture or erosion resulting in thrombus formation [3]. Periodontitis is an inflammatory disease of the tooth supporting tissues caused by specific microorganisms, that leads to progressive destruction of the periodontal ligament and alveolar bone with pocket formation, recession, or both [4]. Different studies have suggested a possible link of atherosclerosis and acute thromboembolic events with chronic dental infections [5, 6]. The probable underlying mechanisms being (a) microbial invasion and infection of atheroma and (b) inflammatory and immunologic mechanisms. Regular introduction of pathogenic oral bacteria into the bloodstream and the elevation of inflammatory mediators have been suggested as possible reasons for increased risk of atherosclerosis and subsequent coronary heart disease [7].

Several studies investigating the connection between periodontitis and acute myocardial infarction (AMI) have shown varying degree of association between them. Periodontitis and CVD have some common characteristics such as higher incidence in adult males, smokers, diabetics, and persons with stress and/or low socioeconomic level. Hence, it is widely believed that the relationship between periodontitis and myocardial infarction is influenced by confounding factors [8]. However, the association between the two parameters exists even after the adjustment of shared risk factors, indicating the independent relationship between periodontitis and AMI [9, 10].

A recent meta‐analysis of cohort studies and other published reviews have shown only moderate level of evidence supporting the causal relationship between periodontal disease and AMI and there is lack of evidence that periodontal interventions reduce the occurrence of AMI, thus, highlighting the necessity of more robust studies to determine the association between the two conditions [11, 12]. No study has been done to determine the association between periodontitis and AMI in Nepalese population. Hence, the aim of this study was to investigate whether there is an association between periodontitis and AMI among the patients visiting a tertiary care center in Koshi Province of Nepal.

## 2. Methods

### 2.1. Study Design and Population

A case–control study was conducted in the Cardiology ward of B.P. Koirala Institute of Health Sciences (BPKIHS), Dharan from 15^th^ August 2022 to 15^th^ March 2023. Patients aged ≥35 years, admitted in the ward with diagnosis of AMI were enrolled as cases. They were diagnosed as per the definition of myocardial infarction by European Society of Cardiology (ESC) as: detection of increase and/or decrease in cardiac markers (preferably troponin) with at least one value above the 99th percentile of the upper reference limit and at least one of the following: symptoms of myocardial ischemia (acute chest discomfort described as pain, pressure, tightness, and burning, chest pain‐equivalent symptoms like dyspnea, epigastric pain, and pain in the left arm), new ischemic ECG changes (persistent or transient ST‐segment elevation, persistent or transient ST‐segment depression, T‐wave inversion, flat T waves, or pseudo‐normalization of T waves), development of pathological Q waves on ECG, imaging evidence of loss of viable myocardium or new regional wall motion abnormality in a pattern consistent with an ischemic etiology or intracoronary thrombus detected on angiography [3]. Controls were recruited from the relatives of the patients, who had no history of AMI in their lifetime and had normal electrocardiogram, after matching for gender and age (±5 years). One control was selected per one case. Those who were physically unstable, those with diagnosis of endocarditis, infectious diseases like hepatitis, tuberculosis, HIV/AIDS, or chronic inflammatory diseases like rheumatoid arthritis and the recipients of periodontal treatment less than 6 months before the examination were not included in this study.

### 2.2. Ethical Approval and Consent

Ethical approval for the study was obtained from the Institutional Review Committee (IRC) of BPKIHS, Dharan (Ref. Number 360/079/080‐IRC). Written informed consent was obtained from all the participants. For those who were illiterate, thumb print was taken and consent form was signed by a witness.

### 2.3. Sample Size Calculation

Sample size for this study was calculated by using the proportions of periodontitis in cases (0.64) and controls (0.29) in a previous study done in Spain by Cueto et al. [9] using software nMaster 2.0 developed by Christian Medical College (CMC), Vellore, India. Considering 80% power of the study and 5% *α* error and adding 20% nonresponse rate, the final sample size required was 37 in each group.

### 2.4. Data Collection Methods

Participants were included in the study after assessing their eligibility and obtaining informed consent. Controls underwent ECG examination to clear them from evidence of myocardial infarction. The participants were interviewed by the investigator using a structured questionnaire, translated and validated in Nepali language, to assess sociodemographic data, tobacco habit, alcohol intake, medical history of hypertension, hypercholesterolemia, and diabetes and family history of CVD. Socioeconomic status was assessed using Kuppuswamy’s socioeconomic scale [13]. Smoking status was assessed as per National Health Interview Survey (NHIS) of Centers for Disease Control and Prevention (CDC) and participants were categorized as current, former and never smokers [14, 15]. Use of smokeless tobacco was assessed using Global Adult Tobacco Survey (GATS) questionnaire and participants were classified as current user, former user, and never user [16]. Based on alcohol consumption, they were classified as lifetime abstainer, former drinker, and current drinker [17, 18].

Medical data of cases about diagnosis of AMI, glucose and cholesterol levels were retrieved from the patient files. For controls, blood samples were collected to measure random blood glucose and cholesterol levels. Blood pressure of all the participants was measured. Height (meter) and weight (kg) of the participants were measured to calculate BMI (kg/m^2^) and was interpreted as underweight (<18.5), healthy weight (18.5–24.9), overweight (25.0–29.9) and obesity (≥30.0). Participants were considered hypertensive if they were under antihypertensive drugs or if they had average systolic blood pressure ≥140 mmHg or diastolic blood pressure ≥90 mmHg in two consecutive measurements taken 1 day apart [19]. Random blood glucose over 200 mg/dL or use of antidiabetic drugs for diagnosed diabetes was the criteria for the participants to be considered as diabetic [20]. Similarly, participants were considered to have hypercholesterolemia if they were under medication for it or if their cholesterol level was higher than 200 mg/dL.

### 2.5. Oral Examination

Oral examination was carried out by a single calibrated examiner. Calibration of the examiner was done in the Department of Public Health Dentistry prior to the study. Reliability test was done in 20% of the sample size, that is, 15 persons. The Kappa value for intraexaminer reliability of the examiner was found to be 0.74 (moderate agreement) for oral hygiene status and 0.88 (strong agreement) for periodontitis.

Examination was done with the cases lying on their bed and controls seated on a chair using LED light. Data were recorded and entered in pro forma by an assistant under direct supervision of the examiner. Cases were examined on the second or third day of admission after ensuring that they were physically stable. Plane mouth mirror and UNC‐15 probe were used to measure probing pocket depth (PD) and clinical attachment loss (AL). Periodontal status was classified as per the case definition proposed by the Centers for Disease Control and Prevention (CDC) in collaboration with the American Academy of Periodontology (AAP)‐CDC/AAP as mild periodontitis (≥2 interproximal sites with AL ≥3 mm, and ≥2 interproximal sites with PD ≥4 mm or one site with PD ≥5 mm), moderate periodontitis (≥2 interproximal sites with AL ≥4 mm (not on same tooth), or ≥2 interproximal sites with PD ≥5 mm), severe periodontitis (≥2 interproximal sites with AL ≥6 mm and ≥1 interproximal site with PD ≥5 mm), or no periodontitis (no evidence of mild, moderate, or severe periodontitis) [21]. Total periodontitis was the sum of mild, moderate, and severe periodontitis. Simplified oral hygiene index (OHI‐S) was used to assess oral hygiene status of the participants. Debris and calculus were measured on buccal or lingual surfaces of six teeth using mouth mirror and explorer. Based on the extent of surface covered by debris and calculus, OHI‐S score (0–6) was calculated and oral hygiene status was categorized as good (0.0–1.2), fair (1.3–3.0), or poor (3.1–6.0) [22].

### 2.6. Data Management and Statistical Analysis

The data collected were entered in Microsoft Excel 365 sheet and exported to Statistical Package for Social Sciences version 20 software (SPSS) for statistical analysis. Mean, standard deviation, percentage and proportion were calculated for descriptive statistics. The association of periodontitis with myocardial infarction was assessed by calculating crude odds ratio (OR) in bivariate logistic regression analysis. For multivariate logistic regression analysis, two models were created. Model I included all the variables from bivariate analysis with *p*  < 0.20, whereas Model II included all the well‐known confounders of the association between periodontitis and AMI that included alcohol consumption, smoking status, use of smokeless tobacco, diabetes mellitus, family history of CVD, cholesterol level, and BMI. The level of significance was set at *p*  < 0.05.

## 3. Results

The study included 74 participants, 37 AMI cases and 37 matched controls. The mean (±SD) age of the participants was 61.9 (±9.8) years. The age of AMI patients ranged from 44 to 79 years. Majority of the participants were male (64.9%). Most of the study individuals (89.2%) were married and belonged to lower socioeconomic class (64.9%). Table 1 shows the association between AMI and sociodemographic characteristics of the study participants. The comparison of the general characteristics of the case and control groups indicated presence of homogeneity between them.

**Table 1:** Association between acute myocardial infarction and sociodemographic characteristics of study participants (*n* = 74).

Variables	Cases, *n* (%)	Controls, *n* (%)	Total, *n* (%)	*p*‐Value ^∗^	Crude OR^a^	95% CI
Age (mean ± SD)	62.3 ± 9.8	61.6 ± 9.9	61.9 ± 9.8	0.747	1.008	0.96–1.06
Gender						
Male	24 (64.9)	24 (64.9)	48 (64.9)	1.00	Ref	—
Female	13 (35.1)	13 (35.1)	26 (35.1)		1.00	0.39–2.59
Marital status						
Married	34 (91.9)	32 (86.5)	66 (89.2)	0.46	Ref	—
Unmarried	3 (8.1)	5 (13.5)	8 (10.8)		0.57	0.13–2.56
Socioeconomic status						
Middle	13 (35.1)	13 (35.1)	26 (35.1)	1.00	Ref	—
Lower	24 (64.9)	24 (64.9)	48 (64.9)		1.00	0.39–2.59

Abbreviations: 95% CI, 95% confidence interval; Ref, reference; SD, standard deviation.

^a^Crude odds ratio.

^∗^Statistical significance *p* < 0.05.

The association of AMI and its risk factors among cases and controls are presented in Table 2. Current smokers showed higher risk of having AMI (OR = 3.00; 95% CI: 0.82–10.99; *p* = 0.097) when compared with never smokers but it was not statistically significant. Current drinkers and former drinkers showed significantly lower odds of having AMI, compared to lifetime abstainers (OR = 0.09; 95% CI: 0.02–0.41; *p* = 0.002 and OR = 0.33; 95% CI: 0.11–0.97; *p* = 0.044, respectively). The mean cholesterol level of the AMI patients was significantly higher compared to the control individuals (OR = 1.02; 95%CI: 1.01–1.03; *p* = 0.009).

**Table 2:** Association between acute myocardial infarction and its risk factors among cases and controls (*n* = 74).

Variables	Cases, *n* (%)	Controls, *n* (%)	Total, *n* (%)	*p*‐Value ^∗^	Crude OR^a^	95% CI
Smoking status						
Never smoker	12 (32.4)	18 (48.6)	30 (40.5)	0.244	Ref	—
Current smoker	10 (27.0)	5 (13.5)	15 (20.3)	0.097	3.00	0.82–10.99
Former smoker	15 (40.5)	14 (37.8)	29 (39.2)	0.367	1.61	0.57–4.50
Usage of smokeless tobacco						
Never user	26 (70.3)	23 (62.2)	49 (66.2)	0.337	Ref	—
Current user	5 (13.5)	10 (27.0)	15 (20.3)	0.187	0.44	0.13–1.49
Former user	6 (16.2)	4 (10.8)	10 (13.5)	0.689	1.33	0.33–5.29
Alcohol consumption						
Lifetime abstainer	22 (59.5)	9 (24.3)	31 (41.9)	**0.005**	Ref	—
Current drinker	3 (8.1)	13 (35.1)	16 (21.6)	**0.002**	0.09	**0.02–0.41**
Former drinker	12 (32.4)	15 (40.5)	27 (36.5)	**0.044**	0.33	**0.11–0.97**
Hypertension						
Absent	15 (40.5)	15 (40.5)	30 (40.5)	1.00	Ref	—
Present	22 (59.5)	22 (59.5)	44 (59.5)		1.00	0.39–2.53
Diabetes mellitus						
Absent	26 (70.3)	28 (75.7)	54 (73.0)	0.601	Ref	—
Present	11 (29.7)	9 (24.3)	20 (27.0)		1.32	0.47–3.69
RBS (mg/dL) (mean ± SD)	142.3 ± 67.6	126.5 ± 43.6	134.4 ± 57.0	0.244	1.005	0.99–1.01
Hypercholesterolemia						
Absent	32 (86.5)	32 (86.5)	64 (86.5)	1.00	Ref	—
Present	5 (13.5)	5 (13.5)	10 (13.5)		1.00	0.26–3.79
Cholesterol level (mg/dL) (mean ± SD)	148.3 ± 39.2	123.7 ± 35.0	136.0 ± 38.9	**0.009**	1.02	**1.01–1.03**
Family history of cardiovascular disease						
No	30 (81.1)	26 (70.3)	56 (75.7)	0.282	Ref	—
Yes	7 (18.9)	11 (29.7)	18 (24.3)		0.55	0.19–1.63
BMI						
Underweight	3 (8.1)	3 (8.1)	6 (8.1)	0.37	Ref	—
Normal weight	24 (64.9)	17 (45.9)	41 (55.4)	0.69	1.41	0.25–7.86
Overweight	9 (24.3)	14 (37.8)	23 (31.1)	0.63	0.64	0.11–3.91
Obese	1 (2.7)	3 (8.1)	4 (5.4)	0.44	0.33	0.02–5.33
BMI score (mean ± SD)	22.7 ± 3.5	24.3 ± 4.3	23.5 ± 3.9	0.079	0.89	0.79–1.01

*Note:* Bold denotes statistically significant.

Abbreviations: 95% CI, 95% confidence interval; BMI, body mass index; RBS, random blood sugar; Ref, reference; SD, standard deviation.

^a^Crude odds ratio.

^∗^Statistical significance *p*  < 0.05.

Table 3 illustrates the association between AMI and oral health variables. Oral hygiene status was similar in both cases and controls. Most of them had fair oral hygiene status (52.9% of cases and 55.6% of controls) and it was not associated with the risk of having AMI. The mean number of teeth present in the control group (27.8 ± 5.4) was higher than that of cases (22.2 ± 9.0) and was significantly associated with AMI (OR = 0.89; 95% CI: 0.82–0.97; *p* = 0.006). Bivariate analysis showed individuals with periodontitis had increased risk of AMI, which was statistically significant (OR = 2.85; 95% CI: 1.08–7.52; *p* = 0.034).

**Table 3:** Association between acute myocardial infarction and oral health variables among cases and controls (*n* = 74).

Variables	Cases, *n* (%)	Controls, *n* (%)	Total, *n* (%)	*p*‐Value^a^	Crude OR^b^	95% CI
OHI‐S score (mean ± SD)	2.9 ± 1.2	2.8 ± 1.0	2.9 ± 1.1	0.636	1.107	0.73–1.69
Oral hygiene status ^∗^						
Good	2 (5.9)	2 (5.6)	4 (5.7)	0.976	Ref	—
Fair	18 (52.9)	20 (55.6)	38 (54.3)	1.00	1.00	0.12–8.13
Poor	14 (41.2)	14 (38.8)	28 (40.0)	0.83	0.90	0.34–2.39
Number of teeth present	22.2 ± 9.0	27.8 ± 5.4	25.0 ± 7.9	**0.006**	0.89	**0.82–0.97**
Periodontitis						
No periodontitis	10 (27.0)	19 (51.4)	29 (39.2)	**0.034**	Ref	—
Periodontitis	27 (73.0)	18 (48.6)	45 (60.8)		2.85	**1.08–7.52**

*Note:* Bold denotes statistically significant.

Abbreviations: 95% CI, 95% confidence interval; OHIS, simplified oral hygiene index; Ref, reference; SD, standard deviation.

^a^Statistical significance *p* < 0.05.

^b^Crude odds ratio.

^∗^Missing data (*n* = 70).

Table 4 presents the results of multivariate analysis for Model I which included alcohol consumption, cholesterol level, BMI score, number of teeth present, and periodontitis. Current alcohol drinkers had significantly lower odds of having AMI compared to lifetime abstainers after adjustment for other variables (OR = 0.12; 95% CI: 0.02–0.62; *p* = 0.011). The significant association between the number of teeth present and AMI seen in bivariate analysis was lost in the multivariate analysis after adjusting for other variables. The association between periodontitis and AMI also diminished after adjusting for other risk factors (adjusted OR = 1.49; 95% CI: 0.45–4.96; *p* = 0.515).

**Table 4:** Logistic regression model for study variables and acute myocardial infarction (Model I).

Variables	ꞵ‐coefficient	*p*‐Value	OR^a^	95% CI
Alcohol consumption				
Lifetime abstainer	—	—	Ref	—
Current drinker	−2.144	**0.011**	0.12	**0.02–0.62**
Former drinker	−1.079	0.101	0.34	0.09–1.24
Cholesterol level	0.015	0.055	1.02	1.00–1.03
BMI score	−1.42	0.076	0.87	0.74–1.02
Number of teeth present	−0.088	0.058	0.92	0.84–1.003
Periodontitis				
No periodontitis	—	0.515	Ref	—
Periodontitis	0.399		1.49	0.45–4.96

*Note:* Model I adjusted for variables with *p*  < 0.20 (alcohol consumption, cholesterol level, BMI score, number of teeth present). Bold denotes statistically significant (*p*  < 0.05).

Abbreviations: 95% CI, 95% confidence interval; BMI, body mass index; Ref, reference.

^a^Adjusted odds ratio.

Table 5 shows the results of Model II logistic regression analysis. The model included variables recognized as classical confounders of the study such as smoking status, smokeless tobacco usage, diabetes mellitus, and family history of CVD in addition to the variables in Model I. Hypertension was not included in this model since there were equal number of hypertensive cases and controls. Model II showed significantly lower odds of having AMI in current and former alcohol drinkers with OR = 0.11; 95% CI: 0.02–0.68; *p* = 0.018 and OR = 0.20; 95% CI: 0.04–0.94; *p* = 0.041, respectively. Mean blood cholesterol level was revealed to be associated with the increased risk of myocardial infarction (OR = 1.02; 95% CI: 1.00–1.04; *p* = 0.041). The independent association between the number of teeth present and risk of myocardial infarction still remained significant after adjustment of other variables (OR = 0.91; 95% CI: 0.82–0.99; *p* = 0.046). The adjusted model showed no association between periodontitis and the risk of myocardial infarction (OR = 1.39; 95% CI: 0.37–5.29; *p* = 0.63).

**Table 5:** Logistic regression model for study variables and acute myocardial infarction (Model II).

Variables	ꞵ‐coefficient	*p*‐Value	OR^a^	95% CI
Alcohol consumption				
Lifetime abstainer	—	—	Ref	—
Current drinker	−2.248	**0.018**	0.11	**0.02–0.68**
Former drinker	−1.602	**0.041**	0.20	**0.04–0.94**
Smoking status				
Never smoker	—	—	Ref	—
Current smoker	1.231	0.190	3.42	0.54–21.58
Former smoker	−0.082	0.918	0.92	0.19–4.39
Usage of smokeless tobacco				
Never user	—	—	Ref	—
Current user	−0.603	0.499	0.55	0.09–3.14
Former user	1.119	0.24	3.06	0.47–19.81
Diabetes mellitus				
Absent	—	—	Ref	—
Present	−0.529	0.486	0.59	0.13–2.61
Family history of cardiovascular disease				
No	—	—	Ref	—
Yes	−0.433	0.582	0.65	0.14–3.03
Cholesterol level	0.019	**0.041**	1.02	1.00–1.04
BMI score	−0.108	0.248	0.89	0.75–1.08
Number of teeth present	−0.099	**0.046**	0.91	**0.82–0.99**
Periodontitis				
No periodontitis	—	—	Ref	—
Periodontitis	0.328	0.630	1.39	0.37–5.29

*Note:* Model II adjusted for well‐known risk factors of acute myocardial infarction (alcohol consumption, smoking status, smokeless tobacco usage, diabetes mellitus, family history of cardiovascular disease, cholesterol level, BMI score). Bold denotes statistically significant (*p*  < 0.05).

Abbreviations: 95% CI, 95% confidence interval; BMI, body mass index; Ref, reference.

^a^Adjusted odds ratio.

## 4. Discussion

AMI is one of the major cardiovascular causes of morbidity and mortality around the globe [23]. Several risk factors have been identified which increase the risk of AMI including age, gender, low‐density lipoprotein, smoking, hypertension, and diabetes mellitus [24]. Recently, periodontitis has also been considered as a potential risk factor for AMI as a huge number of studies have shown a positive association between the two diseases [9, 10, 25–27].

Many studies carried out to investigate the possible causal association between periodontitis and AMI have presented inconsistent findings [9, 20, 26, 28–31]. The present study was conducted to assess the association between chronic periodontitis and AMI among patients admitted in the cardiology ward of BPKIHS with diagnosed AMI. In bivariate analysis, our study showed significant positive association between periodontitis and AMI. Numerous studies back the result of our research [9, 20, 25, 26, 28]. A number of theories have been suggested for the observed association between the two entities. These include hyperreactive monocytes producing large amount of prothrombotic cytokines when stimulated by lipopolysaccharides from gram‐negative oral bacteria, oral bacteria‐induced endothelial damage and increased platelet aggregation, contribution of oral bacteria in thromboembolism during bacteremia and increased levels of leucocytes and fibrinogen caused by periodontal infection that increases blood viscosity and changes blood flow [32]. The strength of the association between periodontitis and AMI observed in the previous studies vary widely with OR ranging from 1.08 to 14.01 [25]. Methodological issues like small study population, varying definitions of periodontitis, and use of data from registries and questionnaires rather than clinical examinations, as well as suboptimal gathering of data on other risk factors, could be the cause of this variability [28].

In the present study, two models were used for regression analysis similar to that done in a previous study [26], that found significant association between periodontitis and AMI in both of the models, which was not the case in our study. The study had shown consistent positive association between periodontitis and AMI across five different case definitions of periodontitis. In our study, even though insignificant association was seen between them, higher proportion of AMI cases had worse periodontal status. Our results are similar to that of Parkar et al. [29], where they found significant association between periodontitis and AMI in bivariate analysis; however, the association was not seen after adjustment of other confounding factors. Other studies have also reported loss of association between the two diseases after adjusting for the common risk factors [30, 31]. These results support the common risk factor hypothesis which is another well‐recognized theory for the observed association between periodontitis and AMI [33]. Syrjänen [34] suggested that both oral infections and vascular diseases result from shared predispositions such as diabetes, smoking, and alcohol consumption, therefore, a direct causal link cannot be established between them. Different prospective cohort studies have reported insignificant independent association between periodontitis and AMI [31, 35]. So, the theory might be true and there may be no causal association between them. However, in past few decades many studies have demonstrated association between periodontitis and myocardial infarction even after consideration of the shared risk factors [9, 26, 32, 36].

In our study, cases and controls were matched for age and gender. The relatives of the patients were enrolled as controls presuming that they share similar risk exposure to the disease. Age is a risk factor for both periodontitis and myocardial infarction [33]. AMI occurs in a wide range of age groups [29], which was seen in this study as the age of cases ranged from 44 to 79 years. The incidence of AMI is more common in males than females [37]. This statement is supported by our study as nearly two‐thirds of the AMI cases were male.

Among various well‐known risk factors of AMI, alcohol consumption was associated with decreased risk of AMI even after adjusting for other variables. The result aligns with the studies of Leong et al. [38] and Schroder et al. [39] that have shown notable protective association of moderate alcohol intake with AMI. Contrary result was shown by some studies [25, 29] where alcohol consumption did not seem to differ among the cases and controls. Possible explanation for the benefit of moderate alcohol consumption are enhanced fibrinolysis and HDL cholesterol, lowered plasma viscosity, fibrinogen levels, platelet aggregation, and inflammatory response, improved endothelial function and antioxidant properties [40]. Excessive drinking can trigger the onset of AMI possibly by showing the opposite effects [41]. Our result should be interpreted with caution since we did not assess the patterns of alcohol consumption in this study. Elevated total cholesterol level is a major risk factor for coronary atherosclerosis [42]. In our study, the cases had significantly higher mean cholesterol level than controls; however, the association between mean cholesterol level and AMI got diminished after adjustment of other factors. Psaty et al. [43] had also reported weak association between total cholesterol level and the risk of myocardial infarction.

Poor oral hygiene is linked not only to an increased risk of AMI but also to sudden cardiac death [44, 45]. Our study found most of the participants had fair or poor oral hygiene and very few had good oral hygiene. This might be partly due to the hospital stay of the AMI patients and their relatives. Though the cases showed slightly worse oral hygiene status, the differences between the two groups was insignificant which conforms to the study results of Zeibolz et al. [46]. In contrast, Parkar et al. [29] and Stein et al. [47] found AMI patients to have poorer oral hygiene compared to the control group. Maintenance of oral hygiene lowers the inflammatory markers, changes risk factors such as diabetes and dyslipidemia, and alters the oral microbiota which ultimately results in a reduced risk of cardiovascular events like AMI and modification of the association between oral health and CVDs [48].

Tooth loss has been used in studies as an indicator for periodontal disease since tooth loss in adults is largely due to periodontitis. However, tooth loss can also result from other causes like dental caries or trauma. Hence, it can merely be regarded as a crude marker of periodontitis [49]. Our study showed association between number of teeth present and the risk of having AMI even after adjusting for the well‐known confounders. Similar result was reported by Kodovazenitis et al. [26], who found significantly higher number of missing teeth in AMI patients. In contrary, Cueto et al. [9] and Lopez et al. [32] demonstrated no significant differences in the number of teeth present in AMI patients and controls.

There are several limitations in this study despite its strength. It was a study conducted at a single center, and the participants were selected using nonrandom sampling technique, which can limit the generalizability of the study results. Moreover, we included AMI patients from the cardiology ward after they had surpassed the critical stage of AMI attack. So technically, they were prevalent cases rather than incident cases. The nonsurvivors could not be included in our study, hence, it cannot be known whether worse periodontal status was associated with survival of AMI patients or not. The information on various variables was obtained using a questionnaire so there is the possibility of information bias or social desirability bias. In addition, radiographic assessment of alveolar bone level could have better represented the periodontal status of the participants, but it was not feasible. The dose–response relationship between periodontitis and AMI was not established. Since the number of participants with mild and severe periodontitis was low, the association of periodontitis severity, and AMI could not be assessed.

## 5. Conclusion

The study revealed that periodontitis was associated with a nonsignificant increased risk for myocardial infarction. AMI patients had worse periodontal status compared to non‐AMI individuals. A high number of missing teeth was significantly related to the risk of having AMI.

Although the causal association between periodontitis and AMI could not be established in this study, the findings have important implications. Large number of AMI patients had periodontitis and greater number of missing teeth denoting poor oral health of the patients. Considering the burden of these two diseases and the findings of our study, preventive strategies addressing both periodontal and cardiovascular risk factors could help mitigate the morbidity and mortality associated with AMI. Proper oral hygiene practices, regular oral health assessment and timely management of periodontal disease can play a pivotal role in lowering the cardiovascular risk. Our study is the first one conducted in Nepalese population, so further studies need to be carried out in different parts of the country including large samples. The inference of lack of association between the two diseases in Nepalese population cannot be simply made based on our study. Further large‐scale case–control studies and cohort studies need to be carried out to fully understand the nature of association between periodontitis and AMI. Randomized controlled trials are also warranted to investigate the effect of periodontal interventions on the reduction of myocardial infarction events.

**Nomenclature**
AMI: Acute myocardial infarction
BPKIHS: B.P. Koirala Institute of Health Sciences
CVD: Cardiovascular diseases
DALY: Disability‐adjusted life years.

## Author Contributions

Binita Limbu and Ashish Shrestha contributed to study conceptualization and design. Data collection was done by Binita Limbu, which was guided by Naveen Kumar Pandey. Statistical analysis were performed by Santosh Kumari Agrawal and Binita Limbu. Manuscript was prepared by Binita Limbu and edited and reviewed by Ashish Shrestha and Tarakant Bhagat.

## Funding

The authors have nothing to report.

## Ethics Statement

Ethical approval for the study was obtained from the Institutional Review Committee (IRC) of BPKIHS, Dharan (Ref. Number 360/079/080‐IRC). Written informed consent was obtained from all the participants. The study followed all the guidelines and regulations of the abovementioned committee (clinical trial number: not applicable).

## Consent

The authors have nothing to report.

## Conflicts of Interest

The authors declare no conflicts of interest.

## Data Availability

The datasets used and/or analyzed in the current study are available from the corresponding author upon reasonable request.
